# Supplementary figures and images for: Transposable element activity captures human pluripotent cell states
Source: EMBO Rep. 2024 Dec 12;26(2):329–52. doi: 10.1038/s44319-024-00343-y (PMC11772670; doi:10.1038/s44319-024-00343-y)

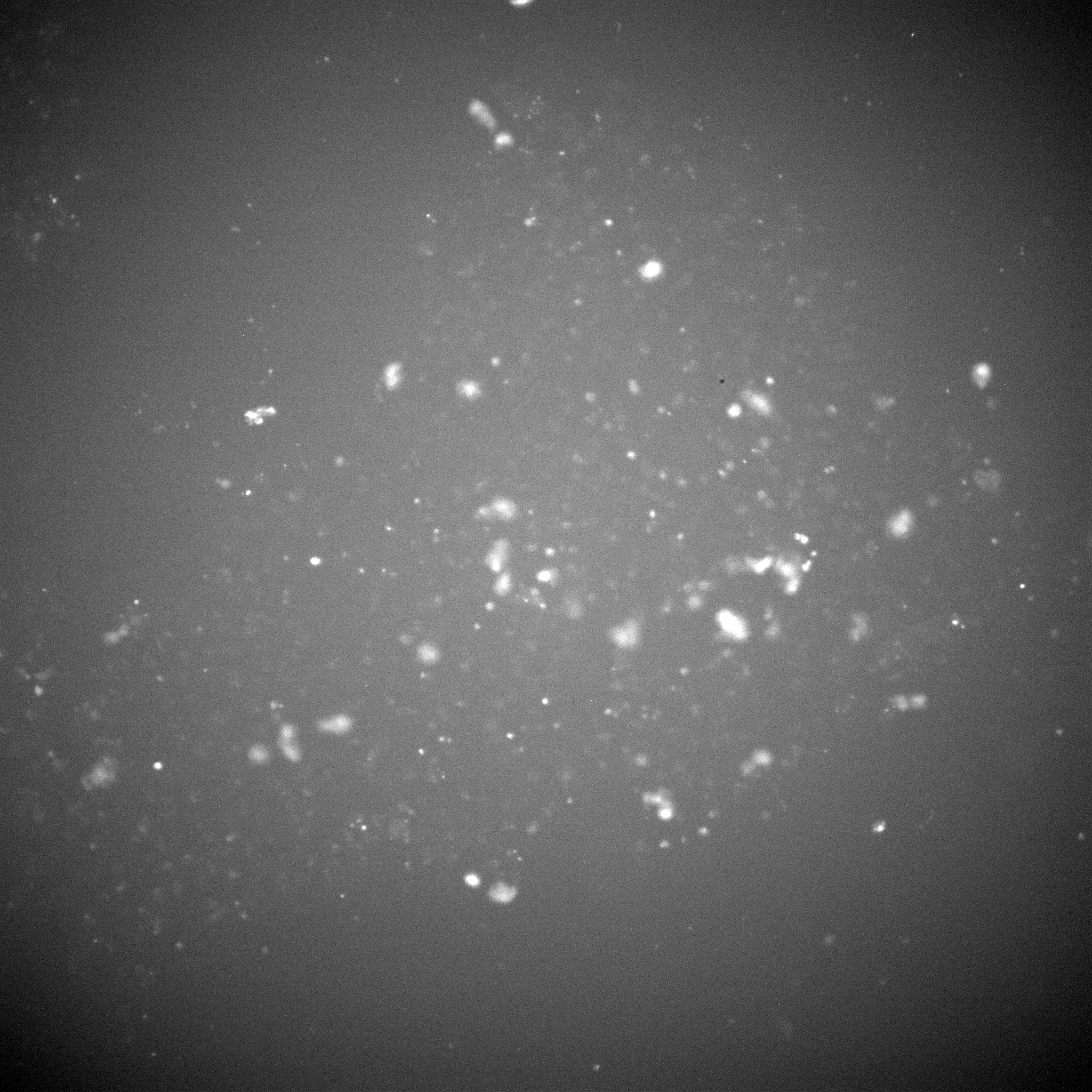

Supplement: Supplementary file 2 — Source data Fig. 1 [file 44319_2024_343_MOESM2_ESM.zip › Figure 1/1C/primed_RFP.tif]

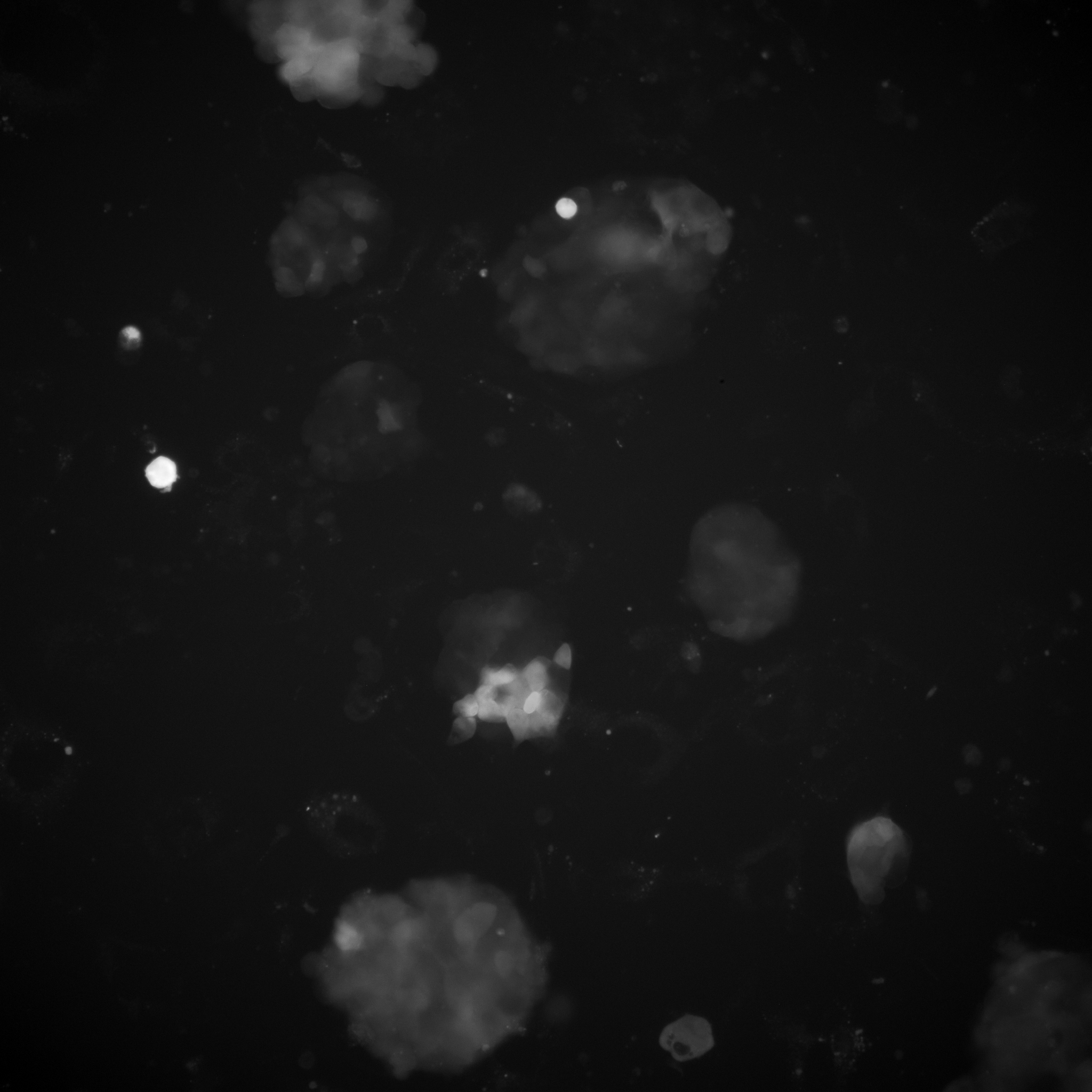

Supplement: Supplementary file 2 — Source data Fig. 1 [file 44319_2024_343_MOESM2_ESM.zip › Figure 1/1C/Naive_GFP.tif]

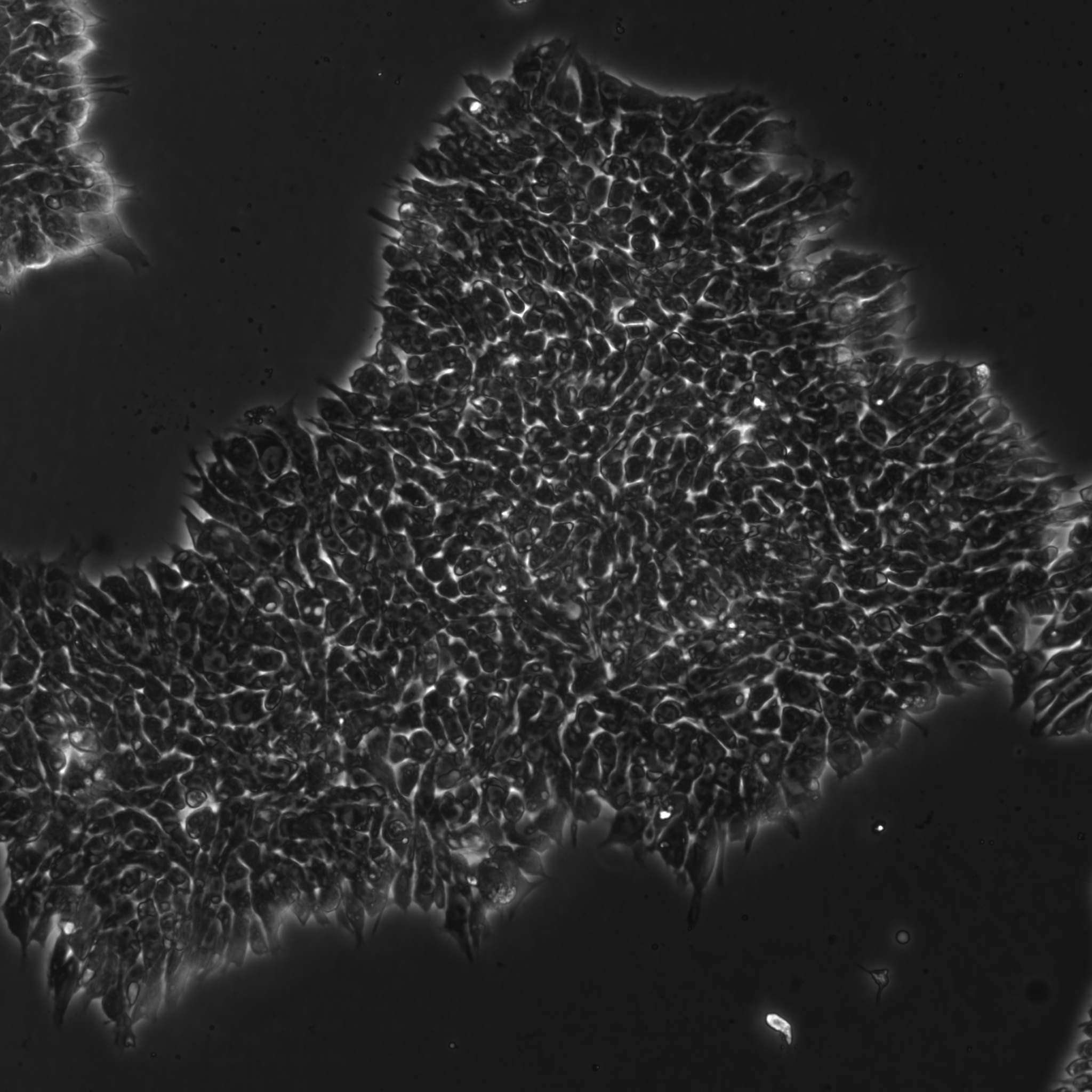

Supplement: Supplementary file 2 — Source data Fig. 1 [file 44319_2024_343_MOESM2_ESM.zip › Figure 1/1C/primed_Bright_field.tif]

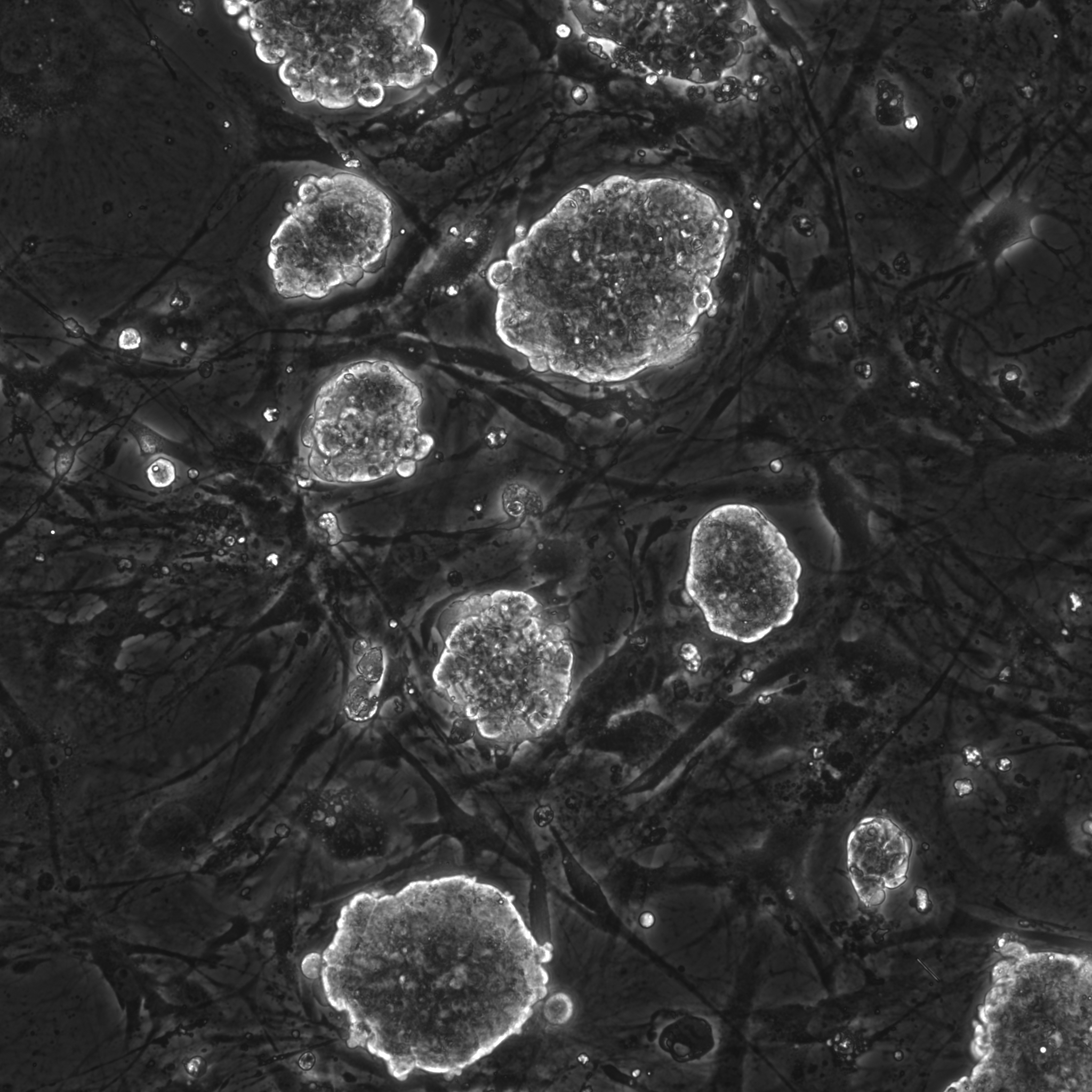

Supplement: Supplementary file 2 — Source data Fig. 1 [file 44319_2024_343_MOESM2_ESM.zip › Figure 1/1C/Naive_Bright_field.tif]

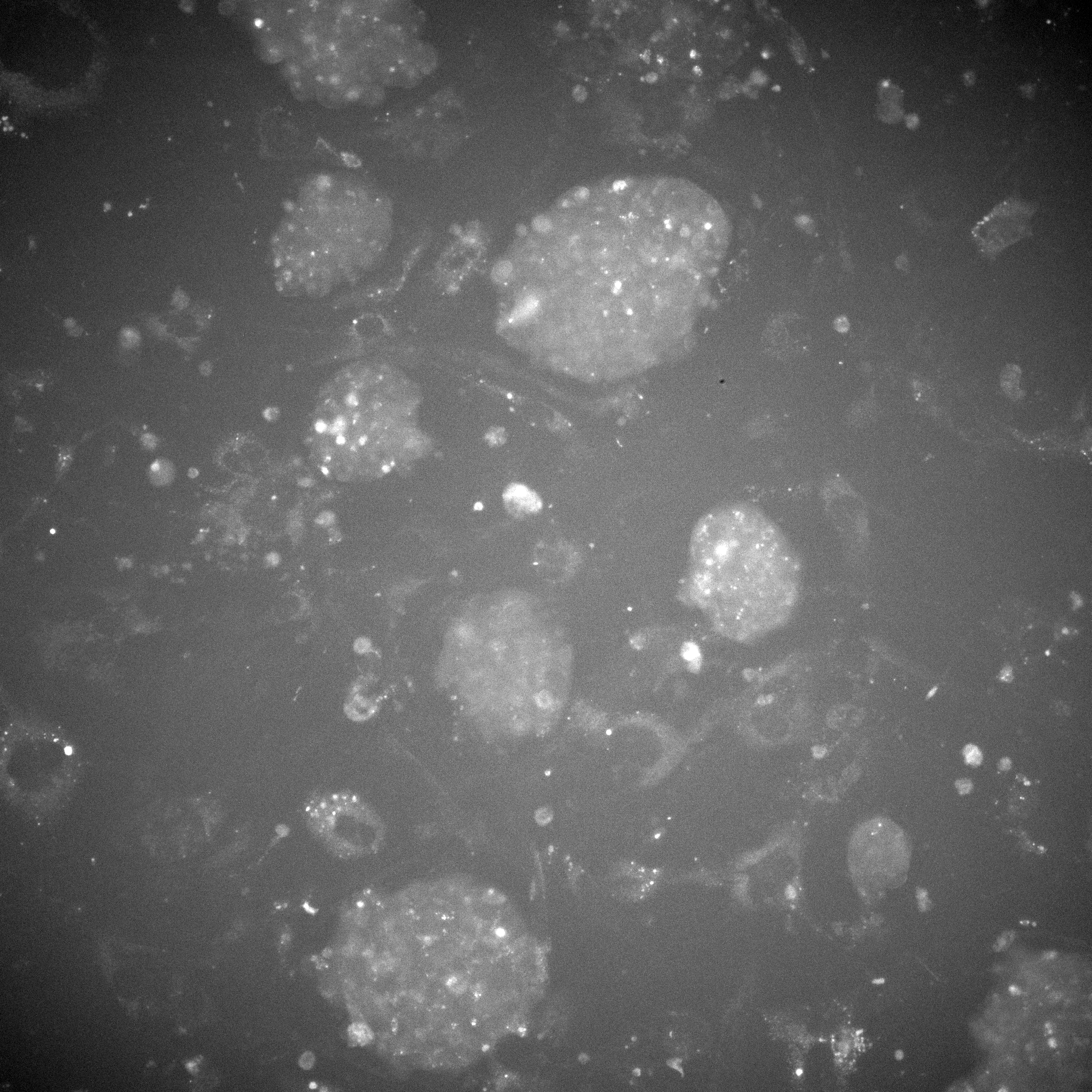

Supplement: Supplementary file 2 — Source data Fig. 1 [file 44319_2024_343_MOESM2_ESM.zip › Figure 1/1C/Naive_RFP.tif]

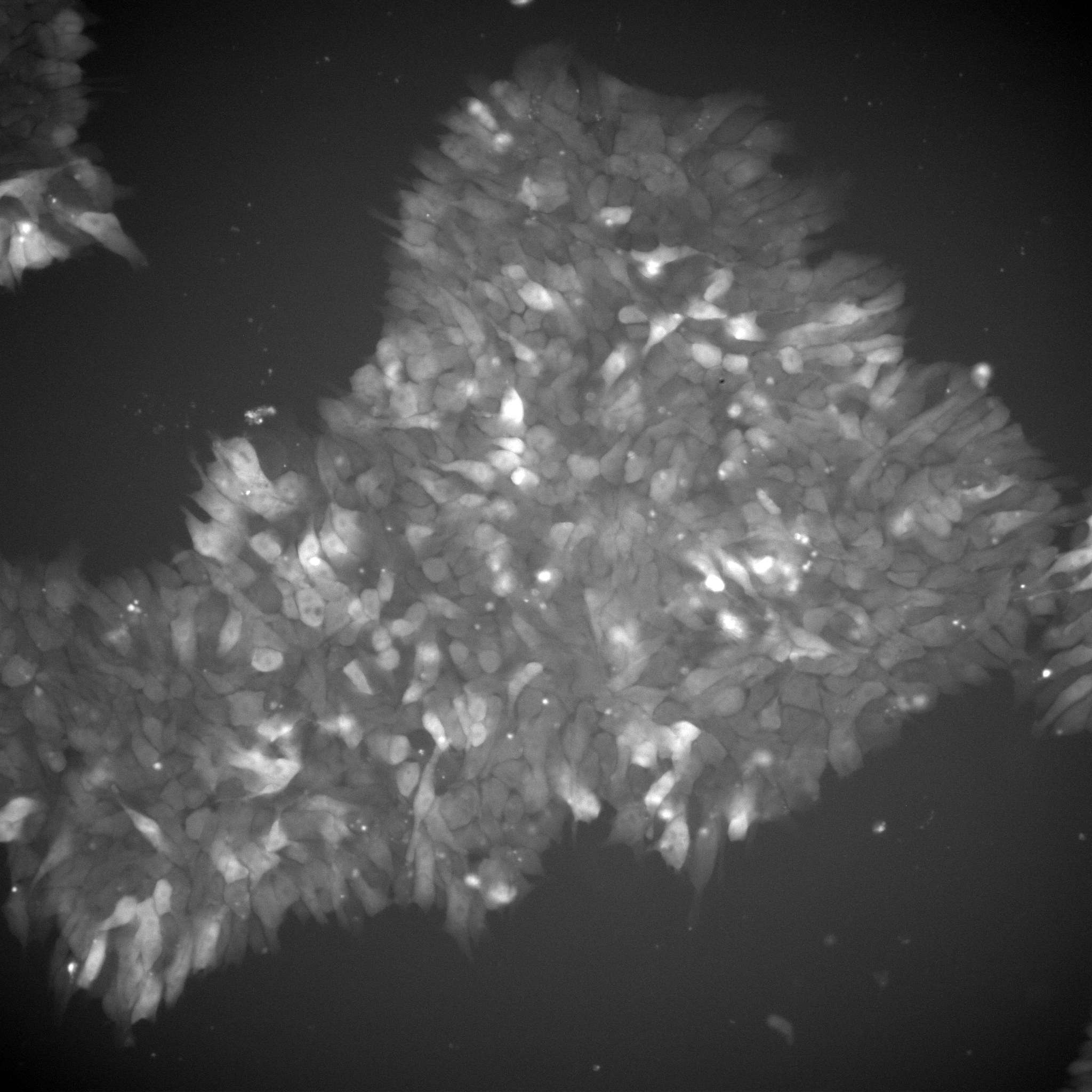

Supplement: Supplementary file 2 — Source data Fig. 1 [file 44319_2024_343_MOESM2_ESM.zip › Figure 1/1C/primed_GFP.tif]

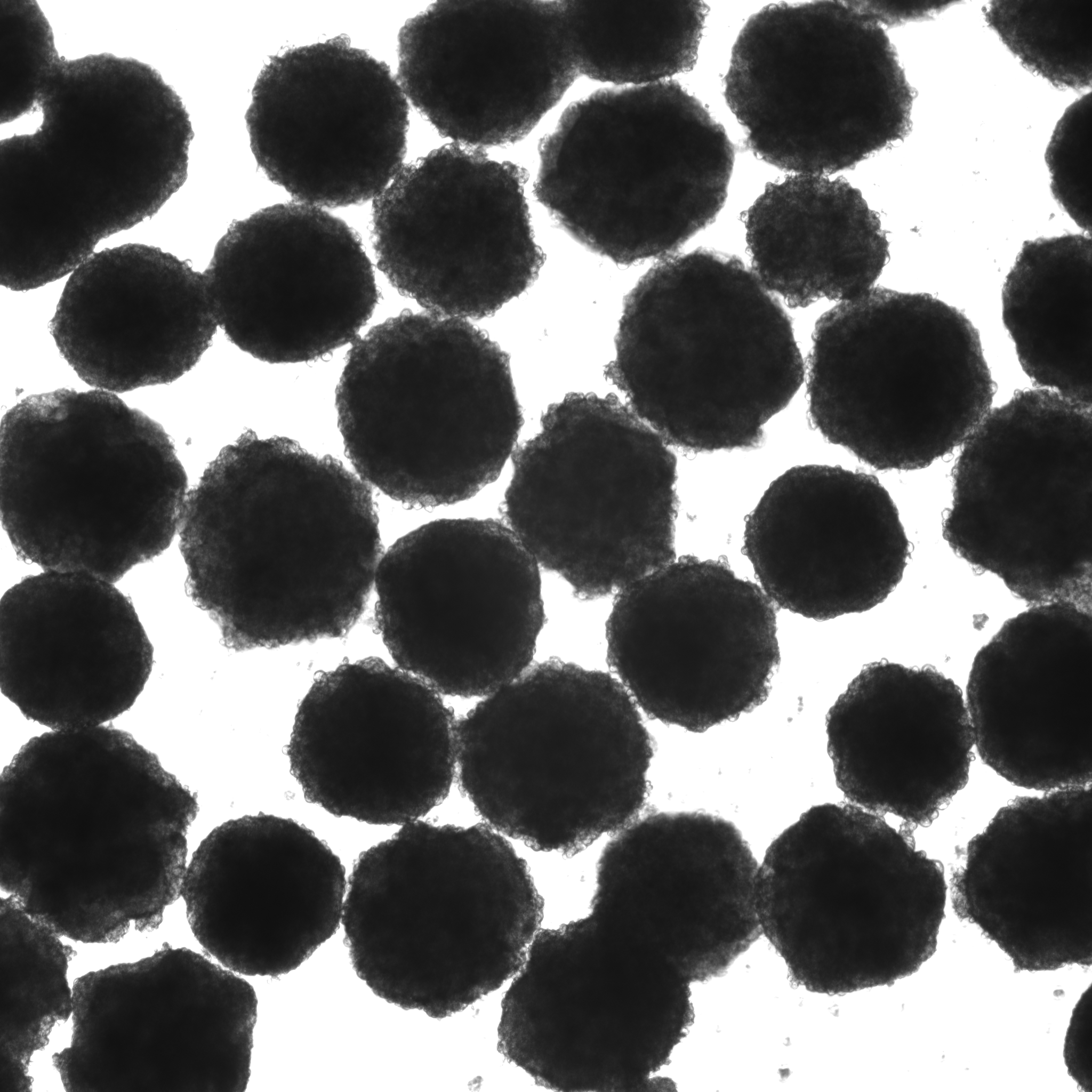

Supplement: Supplementary file 3 — Source data Fig. 2 [file 44319_2024_343_MOESM3_ESM.zip › Figure 2/EV2G/day 4.tif]

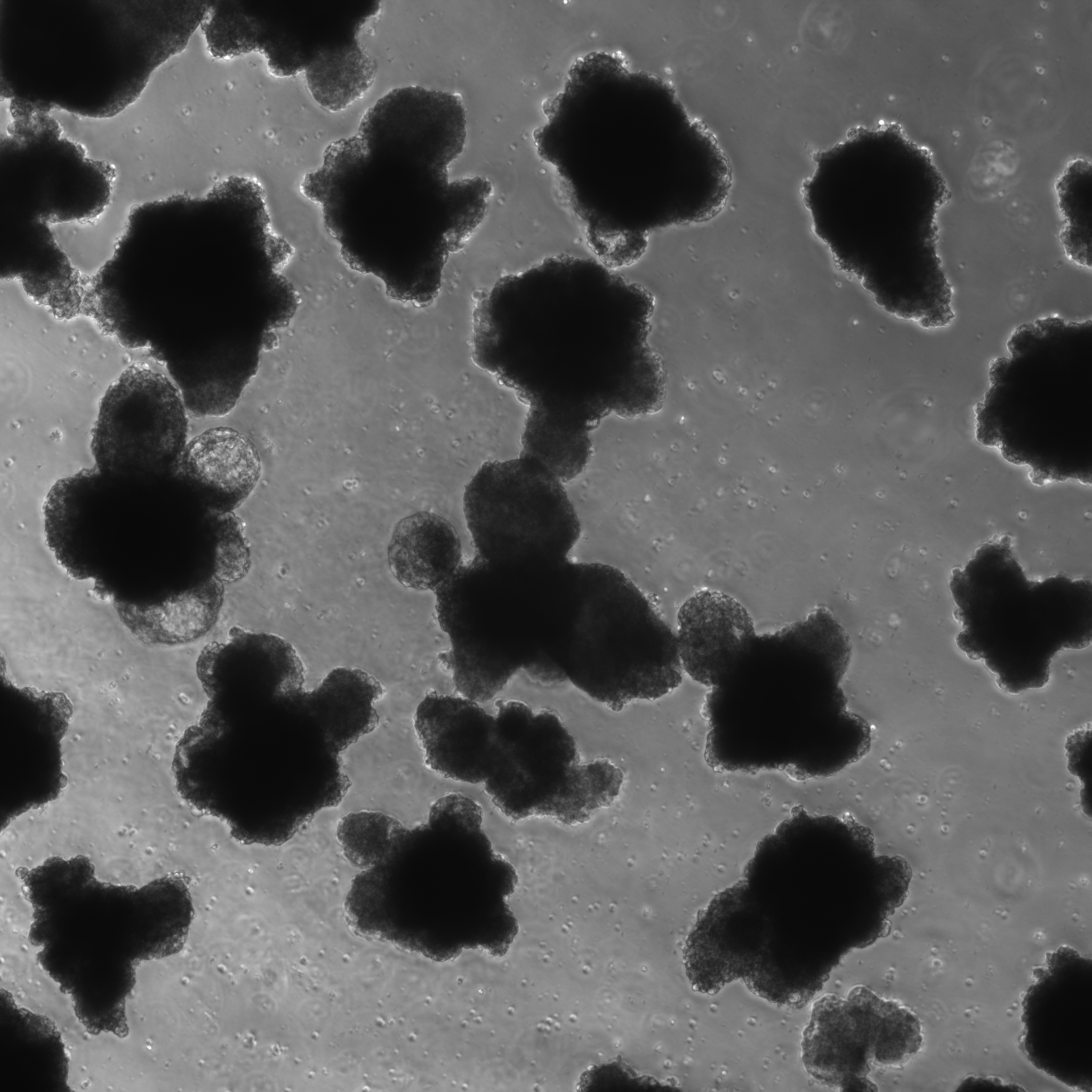

Supplement: Supplementary file 3 — Source data Fig. 2 [file 44319_2024_343_MOESM3_ESM.zip › Figure 2/EV2G/day 12.tif]

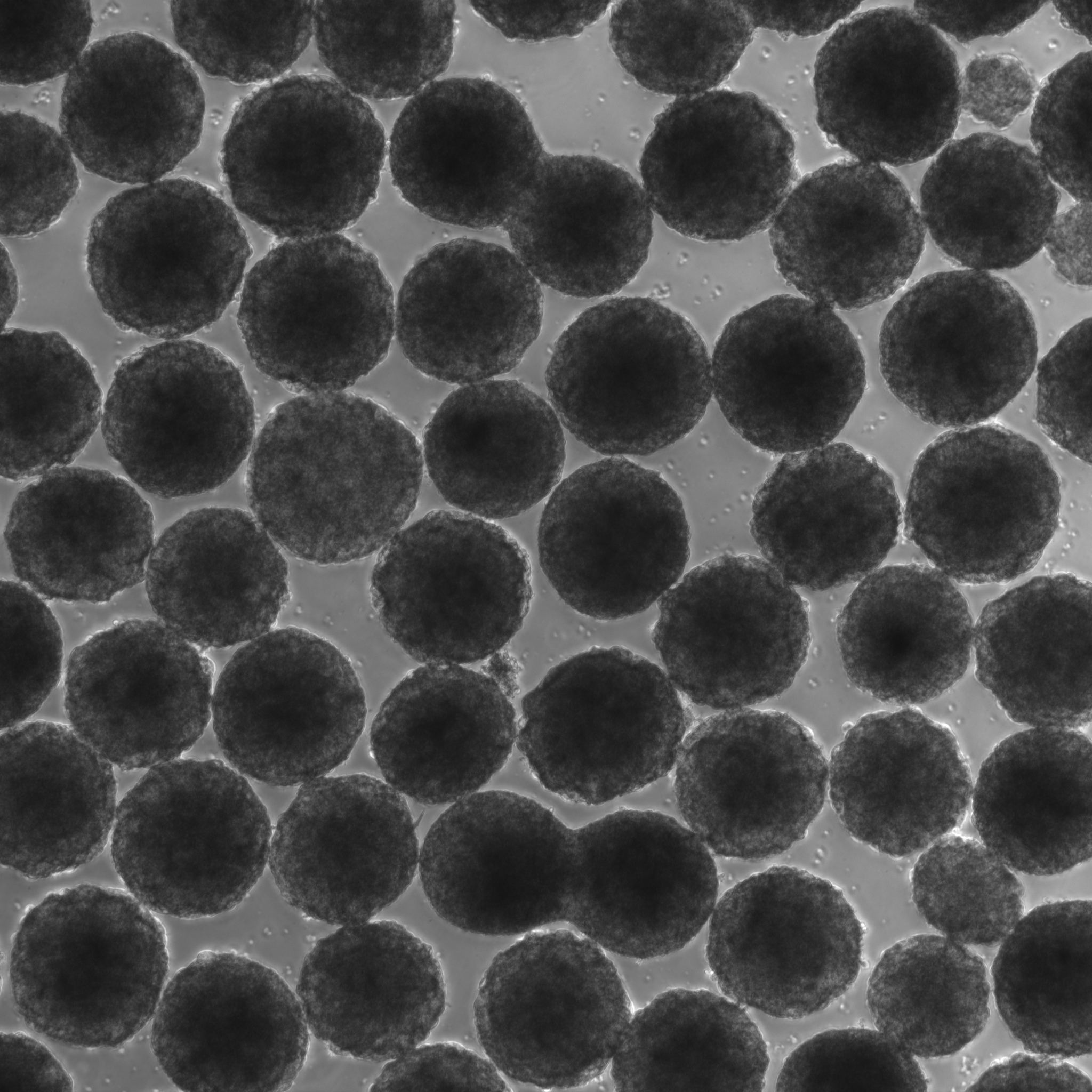

Supplement: Supplementary file 3 — Source data Fig. 2 [file 44319_2024_343_MOESM3_ESM.zip › Figure 2/EV2G/day 0.tif]

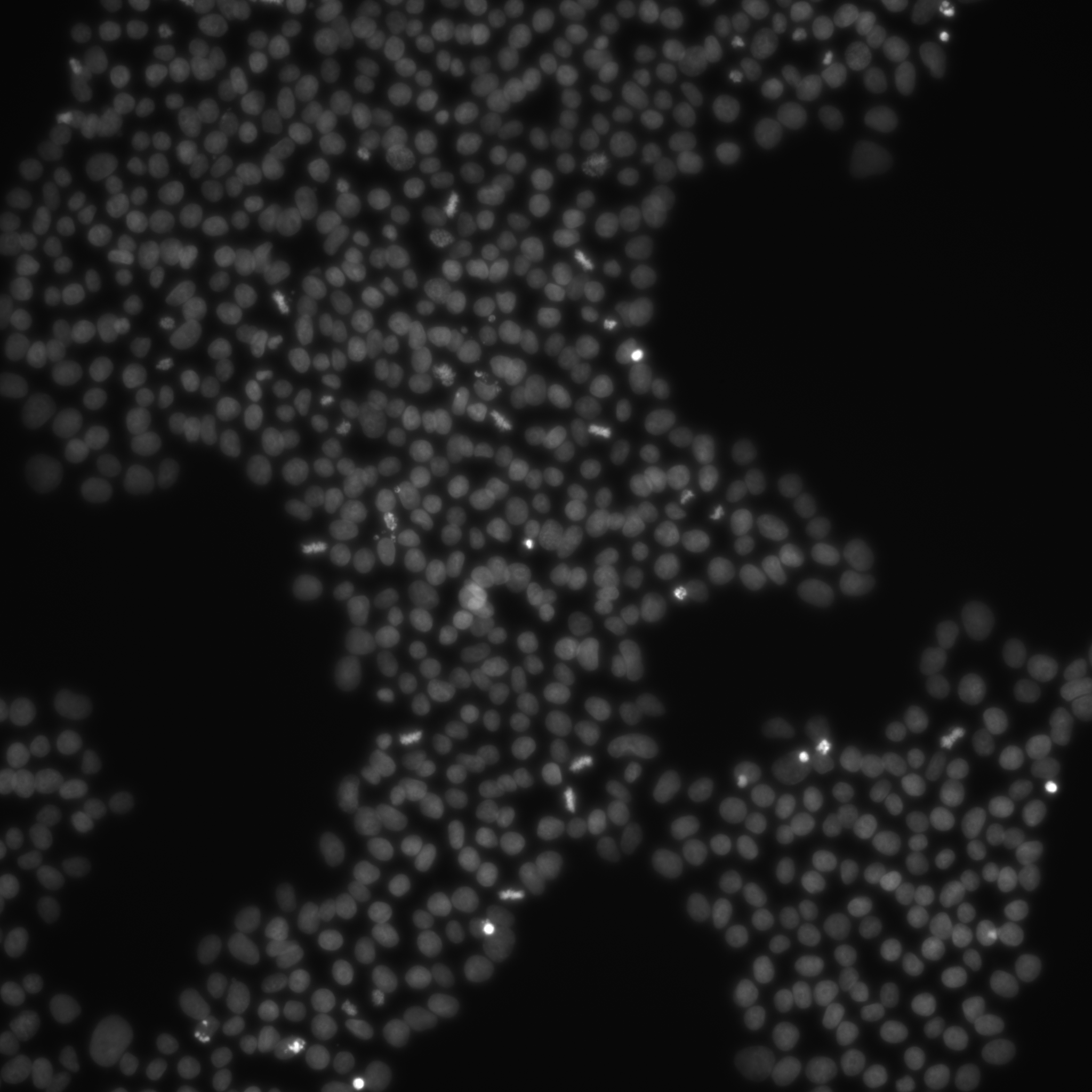

Supplement: Supplementary file 4 — Source data Fig. 3 [file 44319_2024_343_MOESM4_ESM.zip › Figure 3/3M/3M_DAPI.tif]

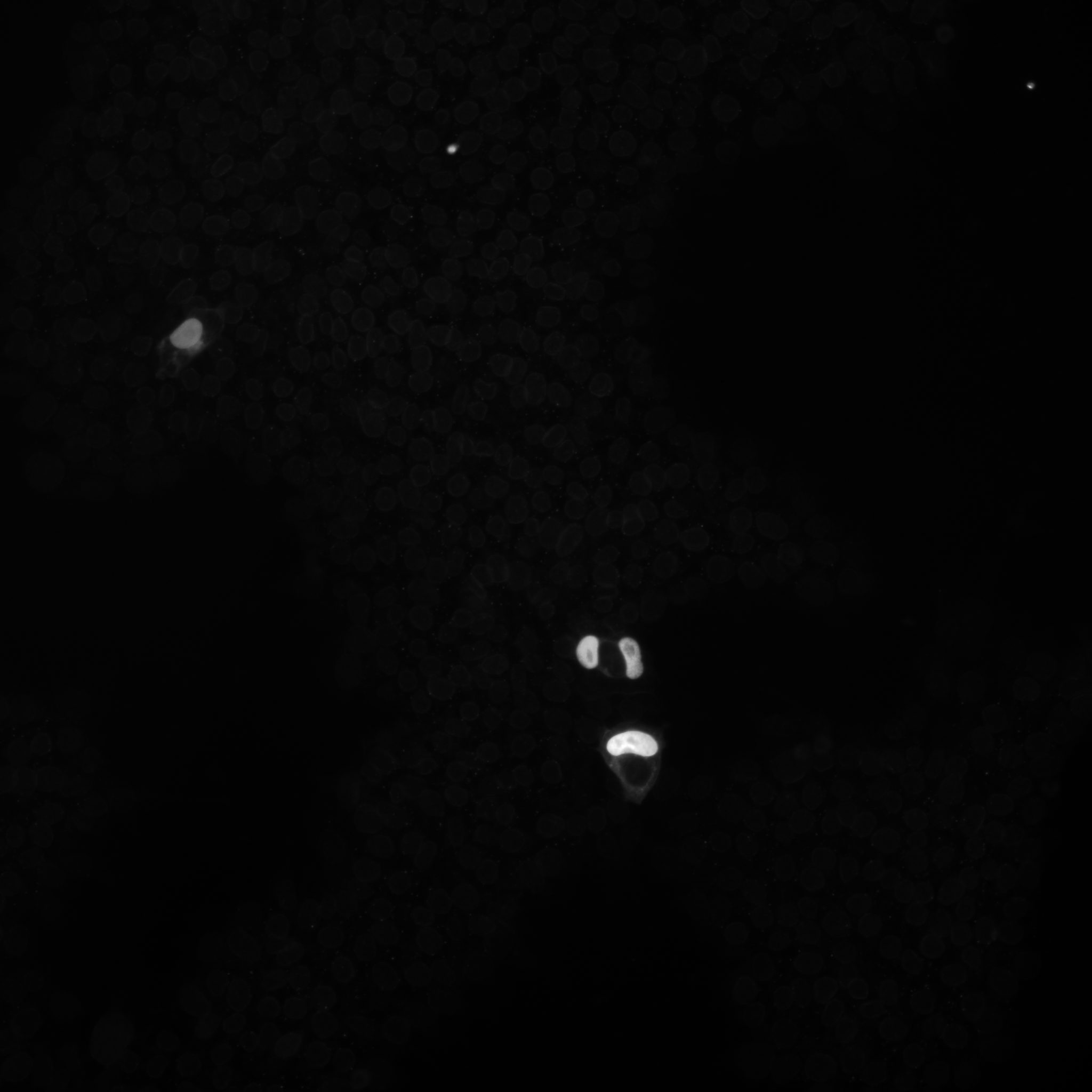

Supplement: Supplementary file 4 — Source data Fig. 3 [file 44319_2024_343_MOESM4_ESM.zip › Figure 3/3M/3M_H3Y.tif]

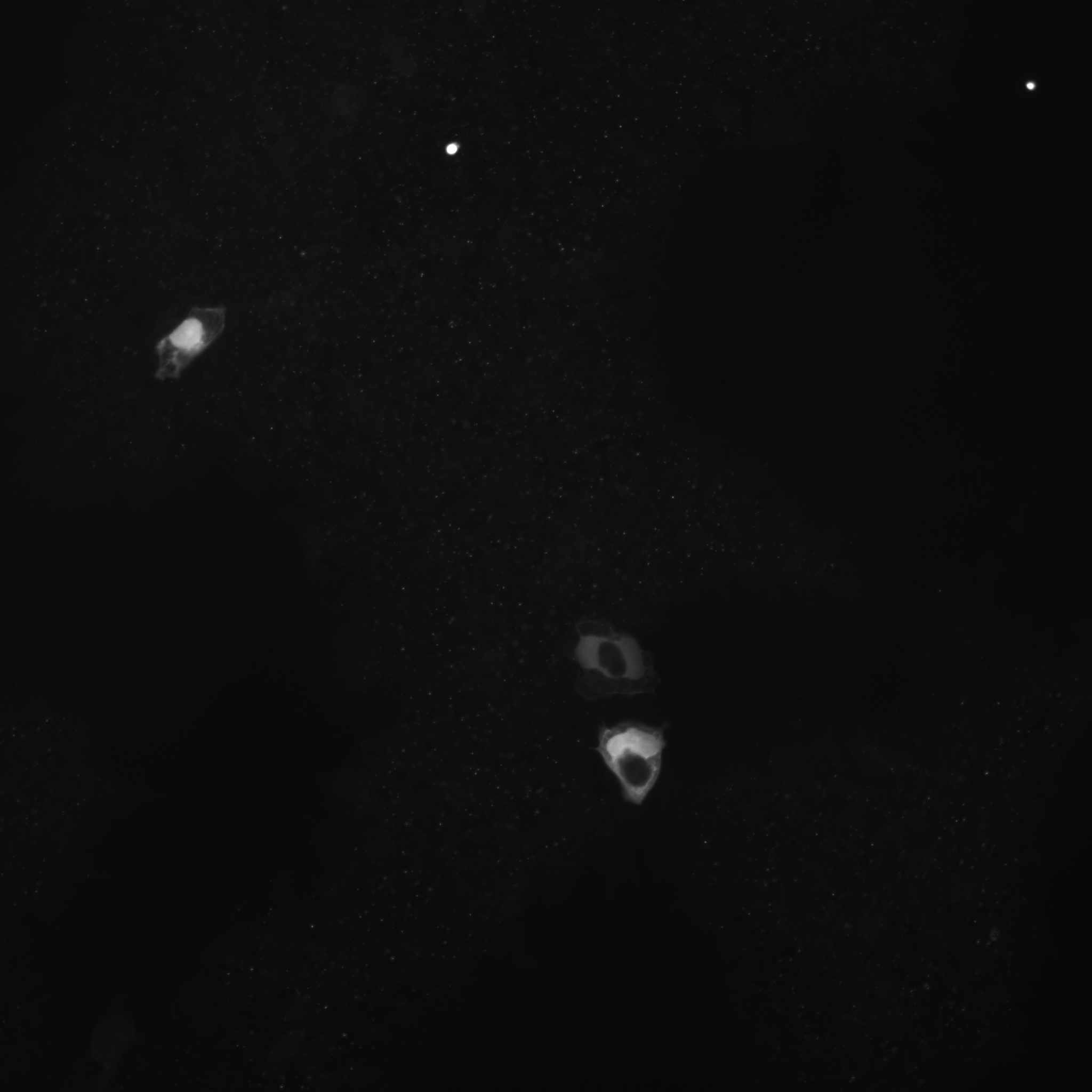

Supplement: Supplementary file 4 — Source data Fig. 3 [file 44319_2024_343_MOESM4_ESM.zip › Figure 3/3M/3M_LTR5_Hs.tif]
