# Supplementary material for: Transposable element activity captures human pluripotent cell states
Source: EMBO Rep. 2024 Dec 12;26(2):329–52. doi: 10.1038/s44319-024-00343-y (PMC11772670; doi:10.1038/s44319-024-00343-y)
Supplement: Supplementary file 6 — Expanded View Figures [file 44319_2024_343_MOESM6_ESM.pdf]

Expanded View Figures

Figure EV1. Differential transcriptional and epigenetic regulation of MER51B and LTR5_Hs in primed and naïve hESCs.

(**A**) Heatmap of RNA-seq data (Data ref: Di Stefano et al, 2018) showing expression of TEs across different hESC lines under primed or conditions. Each column represents an independent biological replicate for primed ($n = 5$) or naïve conditions ($n = 14$). (**B**) Genomic heatmaps (ChIP-seq) showing H3K27ac, NANOG, and OCT4 levels at LTR5_Hs genomic regions in naïve and primed hESCs (Data ref: Chovanec et al, 2021). (**C**) Genomic heatmaps (ChIP-seq) showing SOX2 binding at LTR5_Hs genomic regions in naïve and primed hESCs (Data ref: Chovanec et al, 2021). (**D**) Gene tracks of specific genomic loci for LTR5_Hs and MER51B based on ChIP-seq data.
(**E**) MER51B and LTR5_Hs expression during human preimplantation embryo development (Data ref: Xue et al, 2013). Violin plots show the distribution of read counts for each transposable element across stages of human preimplantation embryo development (zygote: $n = 2$, 2-cell: $n = 3$, 4-cell: $n = 3$, 8-cell: $n = 11$, morula: $n = 3$). The red dotted line indicates the median value, while the black dotted lines represent the interquartile range (IQR), spanning the 25th to 75th percentiles. (**F**) MER51B and LTR5_Hs chromatin accessibility during human preimplantation embryo development (Data ref: Liu et al, 2019). (**G**) Representative phase contrast and fluorescence microscopy images of MER51B-GFP/LTR5_Hs-RFP reporter UCLA4 cells in two different naïve state conditions (5i/LAF and PXGL). Scale bar 100 µm. (**H**) Correlation heatmap showing the Pearson correlation (r) values between UCLA4 MER51B-GFP/LTR5_Hs-RFP primed and naïve hESC RNA-seq samples. The scale bar represents the range of the correlation coefficients (r) displayed.

 © The Author(s)

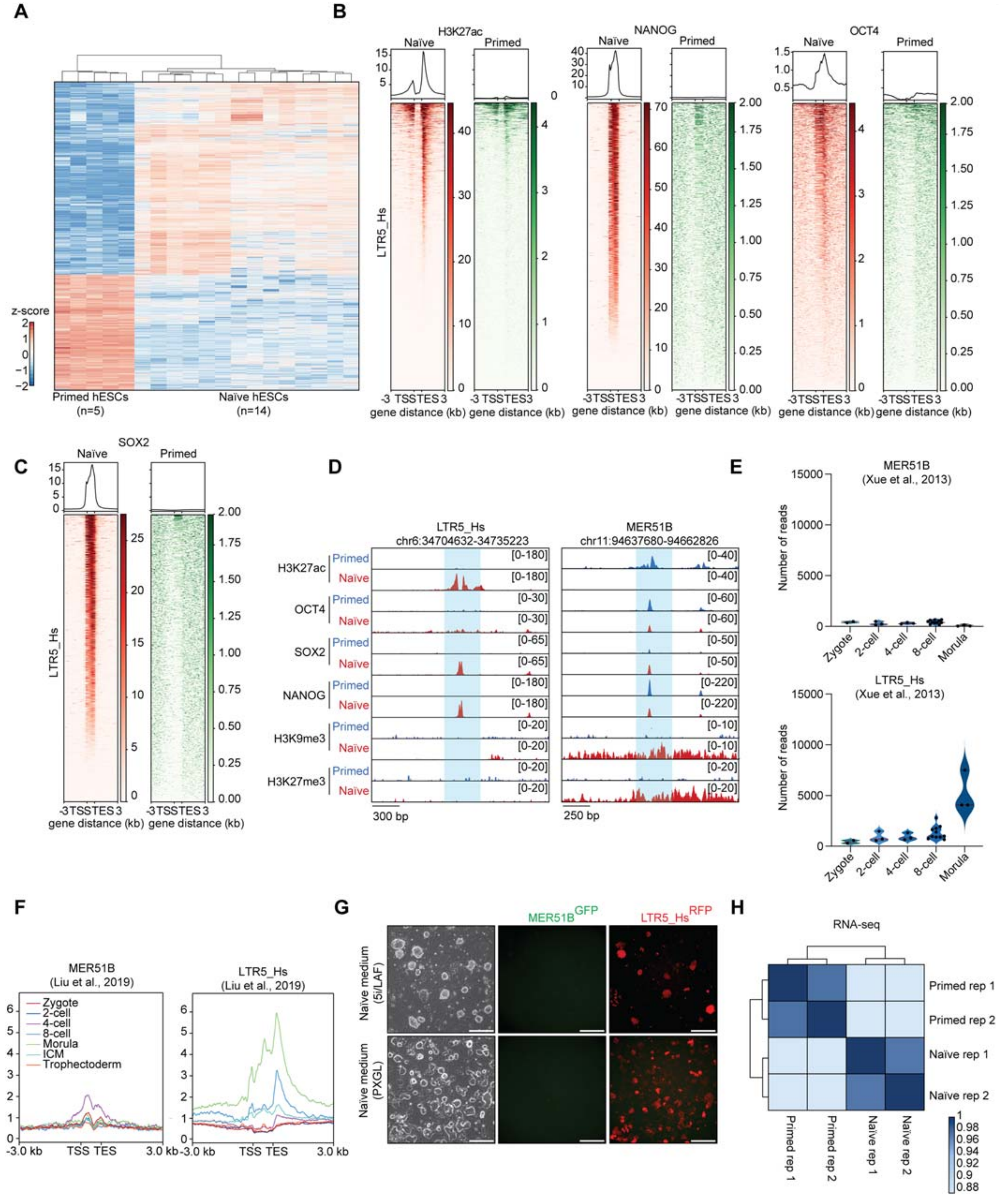
A
z-score
Primed hESCs (n=5)
Naïve hESCs (n=14)
B
H3K27ac
NANOG
OCT4
Naïve
Primed
LTR5_Hs
gene distance (kb)
C
SOX2
D
LTR5_Hs
chr6:34704632-34735223
MER51B
chr11:94637680-94662826
H3K27ac
OCT4
SOX2
NANOG
H3K9me3
H3K27me3
300 bp
250 bp
E
MER51B
(Xue et al., 2013)
LTR5_Hs
(Xue et al., 2013)
Number of reads
Zygote
2-cell
4-cell
8-cell
Morula
F
MER51B
(Liu et al., 2019)
LTR5_Hs
(Liu et al., 2019)
ICM
Trophectoderm
G
MER51BGFP
LTR5_HsRFP
Naïve medium (5i/LAF)
Naïve medium (PXGL)
H
RNA-seq
Primed rep 1
Primed rep 2
Naïve rep 1
Naïve rep 2

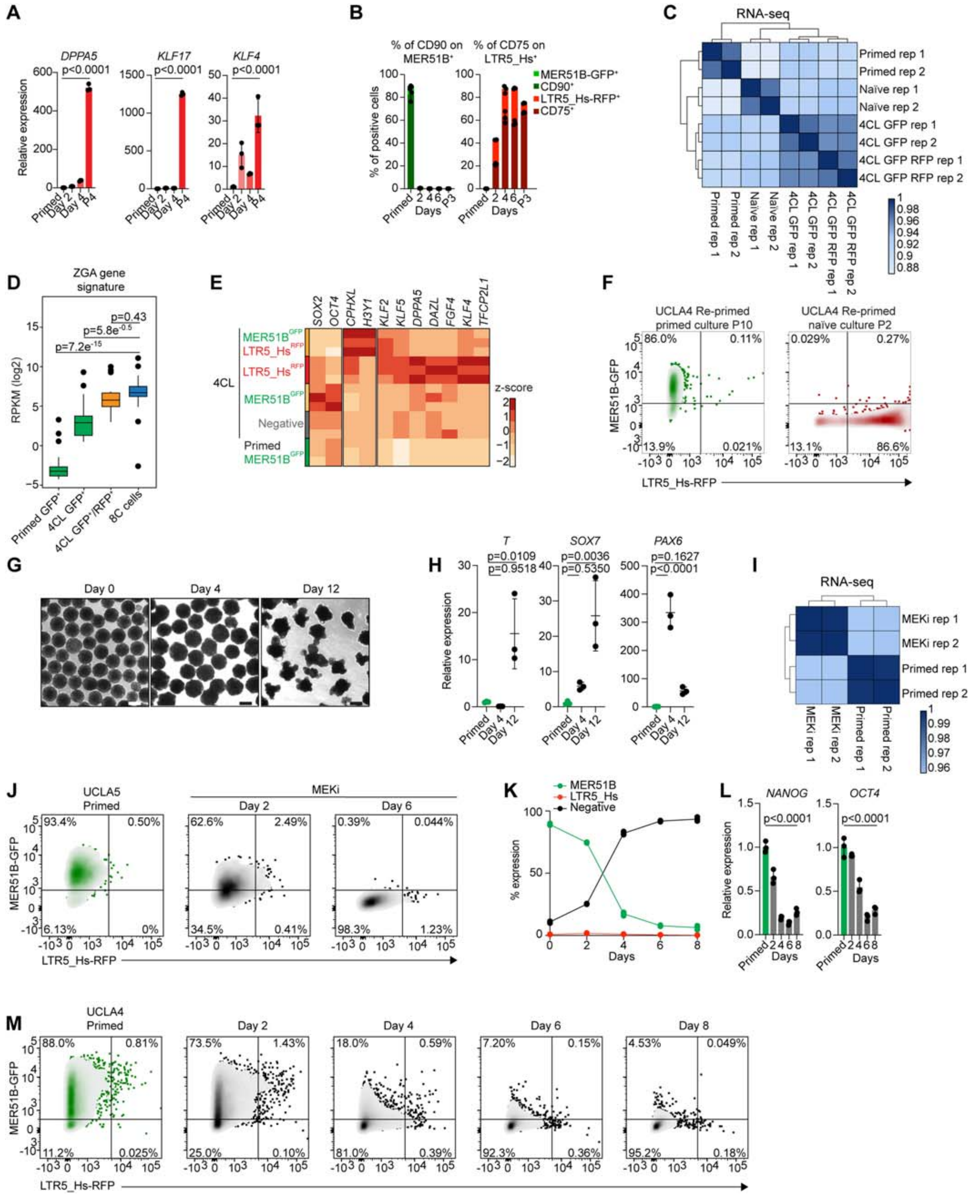
A
DPPA5
KLF17
KLF4
p<0.0001
Relative expression
Primed
Day 2
Day 4
P4
B
% of CD90 on MER51B+
% of CD75 on LTR5_Hs+
% of positive cells
MER51B-GFP+
CD90+
LTR5_Hs-RFP+
CD75+
Days
C
RNA-seq
Primed rep 1
Primed rep 2
Naïve rep 1
Naïve rep 2
4CL GFP rep 1
4CL GFP rep 2
4CL GFP RFP rep 1
4CL GFP RFP rep 2
D
ZGA gene signature
RPKM (log2)
p=0.43
p=5.8e-0.5
p=7.2e-15
Primed GFP+
4CL GFP+
4CL GFP+/RFP+
8C cells
E
SOX2
OCT4
CPHXL
H3Y1
KLF2
KLF5
DPPA5
DAZL
FGF4
KLF4
TFCP2L1
4CL
MER51B GFP
LTR5_Hs RFP
Negative
Primed
z-score
F
UCLA4 Re-primed primed culture P10
UCLA4 Re-primed naïve culture P2
MER51B-GFP
LTR5_Hs-RFP
G
Day 0
Day 4
Day 12
H
T
SOX7
PAX6
p=0.0109
p=0.9518
p=0.0036
p=0.5350
p=0.1627
p<0.0001
Relative expression
I
RNA-seq
MEKi rep 1
MEKi rep 2
Primed rep 1
Primed rep 2
J
UCLA5 Primed
MEKi
Day 2
Day 6
MER51B-GFP
LTR5_Hs-RFP
K
MER51B
LTR5_Hs
Negative
% expression
Days
L
NANOG
OCT4
p<0.0001
Relative expression
Primed
Days
M
UCLA4 Primed
Day 2
Day 4
Day 6
Day 8
MER51B-GFP
LTR5_Hs-RFP

Figure EV2. MER51B and LTR5_Hs activity can be used to monitor hESC conversion and differentiation dynamics.

(**A**) qRT-PCR for the indicated genes during primed-to-naïve conversion at days 2 and 4 and at P4 of an established cell line. Each data point represents an independent biological replicate ($n = 3$). Statistical significance was determined using one-way analysis of variance (ANOVA) with Dunnett's post hoc correction, and exact *p* values are represented in the figure. Data were presented as mean ± standard deviation. Relative gene expression was normalized to *ACTB*. (**B**) Flow cytometric analysis showing the percentage of MER51B-GFP$^+$ cells and CD75 expression in LTR5_Hs-RFP$^+$ cells during the primed-to-naïve transition. Each data point represents an independent biological replicate ($n = 3$). Data were presented as mean ± standard deviation. (**C**) Correlation heatmap showing the Pearson correlation (*r*) values between primed, naïve, and 4CL UCLA4 RNA-seq samples. The scale bar represents the range of the correlation coefficients (*r*) displayed. (**D**) Expression of ZGA ($n = 23$, Taubenschmid-Stowers et al, 2022) genes in primed UCLA4 MER51B-GFP$^+$ cells, 4CL UCLA4 MER51B-GFP$^+$ cells, MER51B-GFP$^+$LTR5_Hs-RFP$^+$ cells, and 8-cell (8C) stage cells (TPRX1$^+$) from (Data ref: Mazid et al, 2022). The box represents the interquartile range (IQR), spanning from the 25th percentile (lower bound) to the 75th percentile (upper bound). The horizontal line within the box indicates the median (50th percentile). Whiskers extend to the smallest and largest values within 1.5 × IQR from the lower (Q1) and upper (Q3) quartiles, respectively. Data points outside this range are plotted as outliers. Statistical significance was determined by unpaired two-tailed Student's *t*-test and exact *p* values are represented in the figure. *P* values >0.05 are non-significant. (**E**) Heatmap showing relative expression values of qRT-PCR for the indicated genes on sorted MER51B-GFP$^+$, MER51B-GFP$^+$LTR5_Hs-RFP$^+$, LTR5_Hs-RFP$^+$, and negative (MER51B-GFP$^-$LTR5_Hs-RFP$^-$) 4CL UCLA4 cells. Each row represents an independent biological replicate ($n = 3$). Relative gene expression was normalized to *HPRT*. (**F**) Representative flow cytometry plots showing changes in MER51B-GFP and LTR5_Hs-RFP expression during primed-to-naïve conversion of reprimed cells shown in Fig. 2F. (**G**) Representative phase contrast images of embryoid bodies at the indicated time points. Scale bar: 100 µm. (**H**) qRT-PCR analysis for *T*, *SOX7*, and *PAX6*, 4 or 12 days after initiating the embryoid body formation assay shown in Fig. 2G, H. Each data point represents an independent biological replicate ($n = 3$). Statistical significance was determined using one-way analysis of variance (ANOVA) with Dunnett's post hoc correction, and exact *p* values are represented in the figure. *P* values >0.05 are non-significant. Data were presented as mean ± standard deviation. Relative gene expression was normalized to *ACTB*. (**I**) Correlation heatmap showing the Pearson correlation (*r*) values between primed and differentiated (MEKi) UCLA4 RNA-seq samples after 6 days. The scale bar represents the range of the correlation coefficients (*r*) displayed. (**J**) Representative flow cytometry plots showing MER51B-GFP and LTR5_Hs-RFP expression during MEKi-induced UCLA5 cell differentiation. (**K**) Flow cytometric quantification of MER51B-GFP and LTR5_Hs-RFP expression during UCLA4 differentiation using primed hESC medium lacking TGF-β and bFGF. Each data point represents an independent biological replicate ($n = 3$). (**L**) qRT-PCR for the indicated genes during the hESC differentiation represented in Fig. EV2K. Each data point represents an independent biological replicate ($n = 3$). Statistical significance was determined using one-way analysis of variance (ANOVA) with Dunnett's post hoc correction, and exact *p* values are represented in the figure. Data were presented as mean ± standard deviation. Relative gene expression was normalized to *ACTB*. (**M**) Representative flow cytometry plots showing MER51B-GFP and LTR5_Hs-RFP expression changes during UCLA4 differentiation using primed hESC medium lacking TGF-β and bFGF.

© The Author(s)

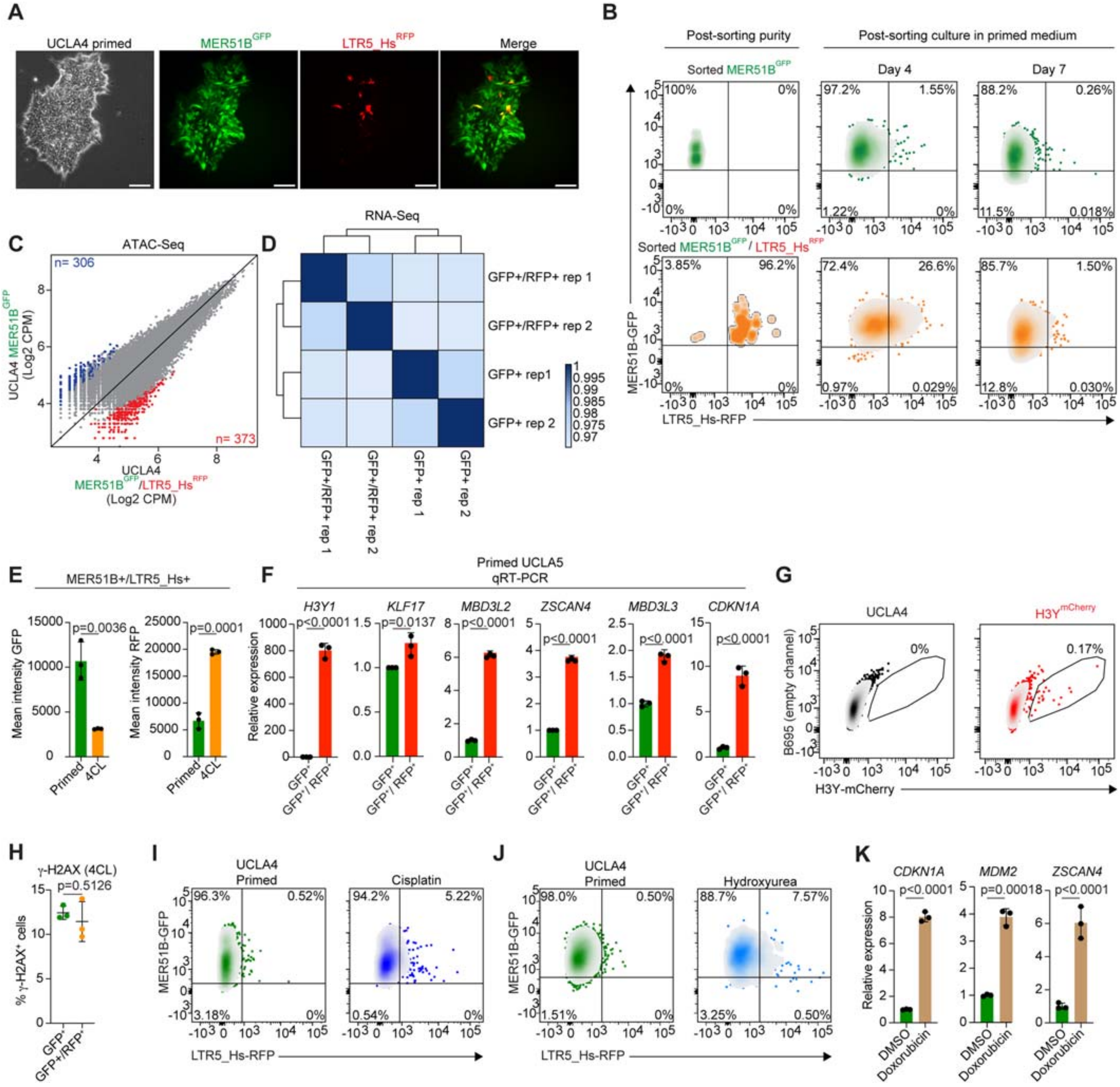
A
UCLA4 primed
MER51B GFP
LTR5_Hs RFP
Merge
B
Post-sorting purity
Post-sorting culture in primed medium
Sorted MER51B GFP
Day 4
Day 7
Sorted MER51B GFP / LTR5_Hs RFP
MER51B-GFP
LTR5_Hs-RFP
C
ATAC-Seq
n= 306
n= 373
UCLA4 MER51B GFP (Log2 CPM)
UCLA4 MER51B GFP/LTR5_Hs RFP (Log2 CPM)
D
RNA-Seq
GFP+/RFP+ rep 1
GFP+/RFP+ rep 2
GFP+ rep1
GFP+ rep 2
E
MER51B+/LTR5_Hs+
p=0.0036
p=0.0001
Mean intensity GFP
Mean intensity RFP
Primed
4CL
F
Primed UCLA5
qRT-PCR
H3Y1
KLF17
MBD3L2
ZSCAN4
MBD3L3
CDKN1A
p<0.0001
p=0.0137
Relative expression
G
UCLA4
H3Y mCherry
B695 (empty channel)
H3Y-mCherry
H
γ-H2AX (4CL)
p=0.5126
% γ-H2AX+ cells
I
UCLA4 Primed
Cisplatin
J
Hydroxyurea
K
CDKN1A
MDM2
ZSCAN4
p=0.00018
DMSO
Doxorubicin

Figure EV3. MER51B-GFP and LTR5_Hs-RFP label a primed, metastable hESC population that expresses ZGA and DNA damage response genes.

(**A**) Representative phase contrast and fluorescence microscopy images of UCLA4 MER51B-GFP/LTR5_Hs-RFP hESCs showing rare GFP^+RFP^+ (yellow) cells amongst the primed cell population. Scale bar: 100 µm. (**B**) Representative flow cytometry plots showing reporter expression changes in sorted MER51B-GFP^+ and MER51B-GFP^+LTR5_Hs-RFP^+ UCLA4 cells at the indicated time points after culture. (**C**) Scatterplot showing ATAC-seq analysis of chromatin accessibility for MER51B-GFP^+ and MER51B-GFP^+LTR5_Hs-RFP^+ UCLA4 cells. Two ($n = 2$) independent biological replicates per group were analyzed. Blue dots indicate genomic regions showing significantly decreased chromatin accessibility in MER51B-GFP^+LTR5_Hs-RFP^+ cells (Log2FC <−1.5, $p < 0.05$, $n = 306$); red dots indicate genomic regions showing significantly increased chromatin accessibility in MER51B-GFP^+LTR5_Hs-RFP^+ cells (Log2FC >1.5, $p < 0.05$, $n = 373$). (**D**) Correlation heatmap showing the Pearson correlation (r) values between UCLA4 MER51B-GFP^+ and MER51B-GFP^+LTR5_Hs-RFP^+ RNA-seq samples. The scale bar represents the range of the correlation coefficients (r) displayed. (**E**) Mean intensity of MER51B-GFP and LTR5_Hs-RFP on gated MER51B-GFP^+LTR5_Hs-RFP^+ cells in primed versus 4CL condition. Each data point represents an independent biological replicate ($n = 3$). Statistical significance was determined by unpaired two-tailed Student's *t*-test, and exact *p* values are represented in the figure. Data were presented as mean ± standard deviation. (**F**) qRT-PCR for the indicated genes in primed MER51B-GFP^+LTR5_Hs-RFP^+ UCLA5 cells. Each data point represents an independent biological replicate ($n = 3$). Statistical significance was determined by unpaired two-tailed Student's *t*-test, and exact *p* values are represented in the figure. Data were presented as mean ± standard deviation. Relative gene expression was normalized to *ACTB*. (**G**) Representative flow cytometry plots showing basal H3Y-mCherry expression in primed UCLA4 cells. (**H**) Flow cytometric quantification for the percentage of γ-$H2AX^+$ cells in gated MER51B-GFP^+LTR5_Hs-RFP^+ versus MER51B-GFP^+ UCLA4 cells cultured in 4CL. Each data point represents an independent biological replicate ($n = 3$). Statistical significance was determined by unpaired two-tailed Student's *t*-test, and exact *p* values are represented in the figure. *P* values >0.05 are non-significant. Data were presented as mean ± standard deviation. (**I**) Representative flow cytometry plots showing primed MER51B-GFP^+/LTR5_Hs-RFP^+ cells 40 h after washing off DMSO or cisplatin treatment (treatment length: 10 h). (**J**) Representative flow cytometry plots showing primed MER51B-GFP/LTR5_Hs-RFP cells 12 h after washing off DMSO or hydroxyurea treatment (treatment length: 12 h). (**K**) qRT-PCR for the indicated genes in bulk-primed cells treated with doxorubicin for 8 h. Each data point represents an independent biological replicate ($n = 3$). Statistical significance was determined by unpaired two-tailed Student's *t*-test, and exact *p* values are represented in the figure. Data were presented as mean ± standard deviation. Relative gene expression was normalized to *ACTB*.

© The Author(s)

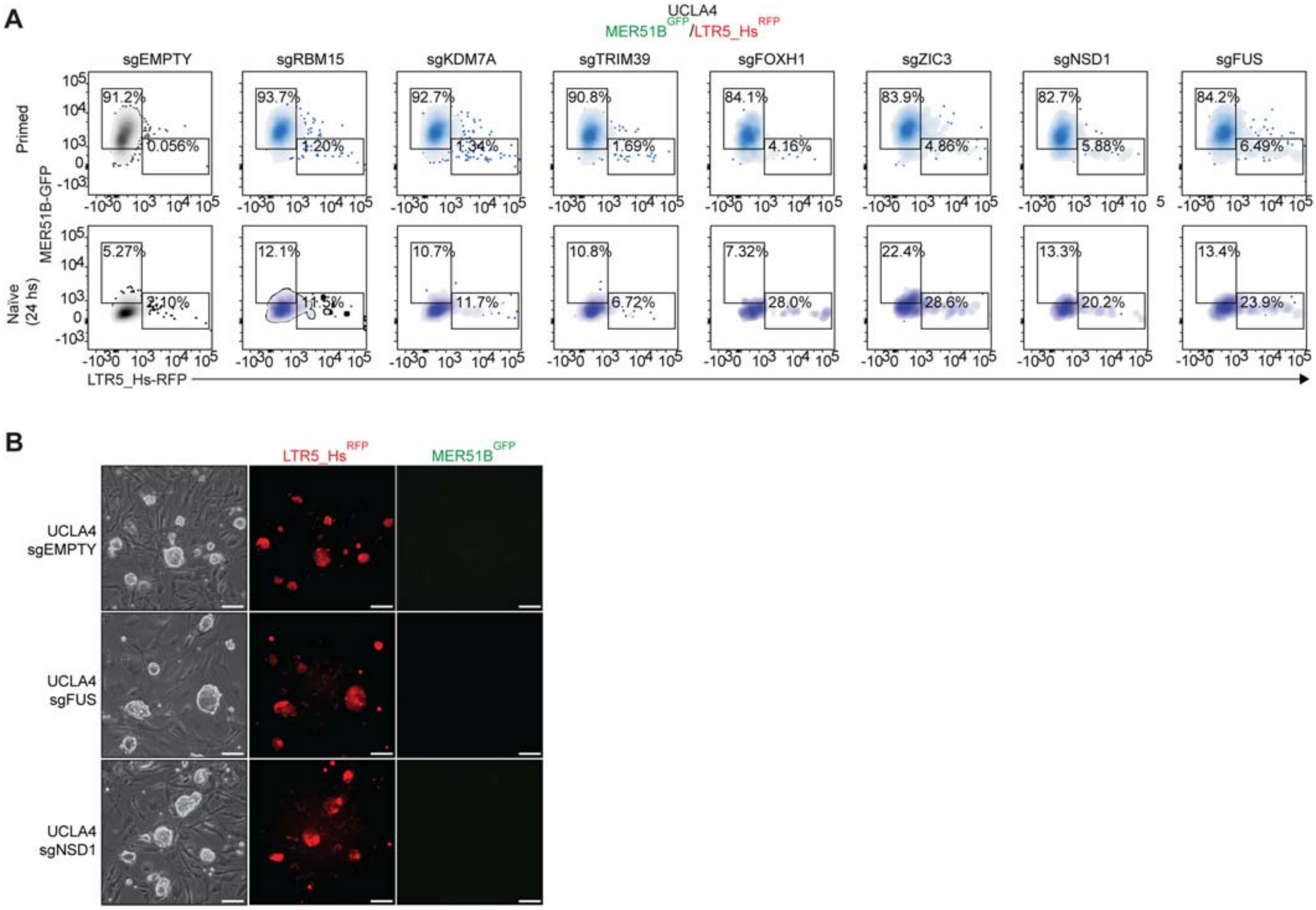

Figure EV4. Use of MER51B and LTR5_Hs dual reporter system to study regulators of stem cell potency.

(**A**) Representative flow cytometry plots showing changes in MER51B-GFP and LTR5_Hs-RFP expression after knockout of the indicated genes, in primed medium or naïve culture conditions for 24 h. (**B**) Representative phase contrast and fluorescence microscopy images of established naïve cell cultures (P3) of UCLA4 MER51B-GFP/LTR5_Hs-RFP hESC after knockout of FUS and NSD1. Scale bar: 300 μm.

© The Author(s)
